# Aconitic Acid Recovery from Renewable Feedstock and Review of Chemical and Biological Applications

**DOI:** 10.3390/foods11040573

**Published:** 2022-02-16

**Authors:** Gillian O. Bruni, K. Thomas Klasson

**Affiliations:** U.S. Department of Agriculture, Agricultural Research Service, New Orleans, LA 70124, USA; thomas.klasson@usda.gov

**Keywords:** aconitic acid, polyester, polymer, tissue engineering, plasticizer, cross-linker, antifeedant, sugarcane, sweet sorghum, molasses

## Abstract

Aconitic acid (propene-1,2,3-tricarboxylic acid) is the most prevalent 6-carbon organic acid that accumulates in sugarcane and sweet sorghum. As a top value-added chemical, aconitic acid may function as a chemical precursor or intermediate for high-value downstream industrial and biological applications. These downstream applications include use as a bio-based plasticizer, cross-linker, and the formation of valuable and multi-functional polyesters that have also been used in tissue engineering. Aconitic acid also plays various biological roles within cells as an intermediate in the tricarboxylic acid cycle and in conferring unique survival advantages to some plants as an antifeedant, antifungal, and means of storing fixed pools of carbon. Aconitic acid has also been reported as a fermentation inhibitor, anti-inflammatory, and a potential nematicide. Since aconitic acid can be sustainably sourced from renewable, inexpensive sources such as sugarcane, molasses, and sweet sorghum syrup, there is enormous potential to provide multiple streams of additional income to the sugar industry through downstream industrial and biological applications that we discuss in this review.

## 1. Introduction

**Figure 1:**
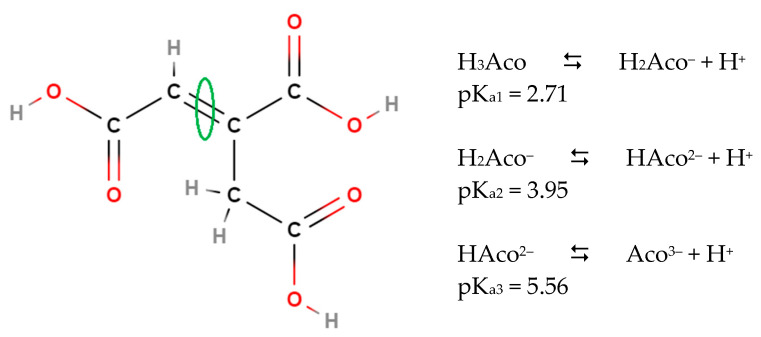
Chemical structure of *trans*-aconitic acid and its three stepwise carboxylic acid dissociation constants in aqueous solution. *Cis*-aconitic acid is obtained by rotation around the circled double bond. Dissociation constants [3,4].

Aconitic acid, propene-1,2,3-tricarboxylic acid (Figure 1), is listed as one of the top 30 value-added chemicals by the Department of Energy for its use in industry as a chemical building block and precursor to other important chemicals and polymers [1]. Aconitic acid also plays a role in biological systems and has numerous applications that will be discussed herein [2].

**Figure 2:**
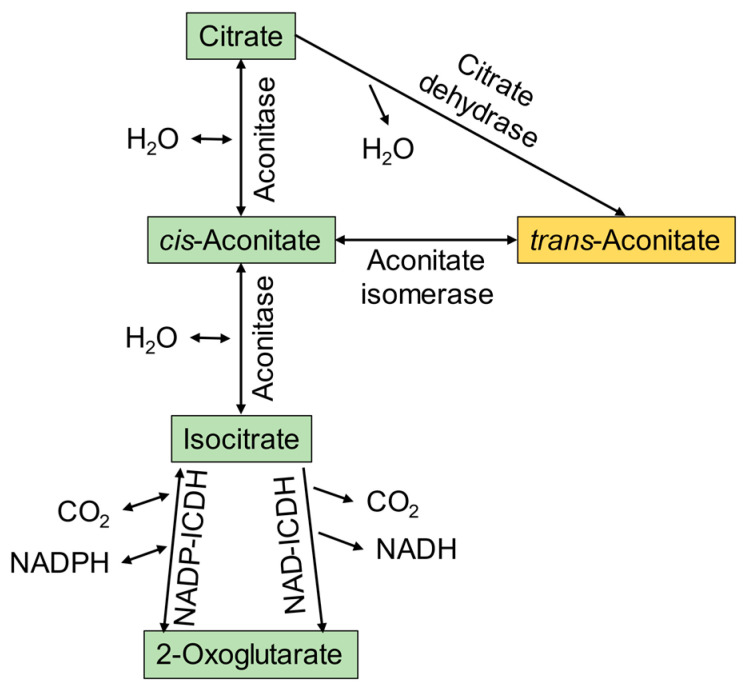
Citrate branch of the TCA cycle. Two separate enzymes result in the formation of *trans*-aconitate via (1) aconitase-mediated conversion of *cis*- to *trans*-aconitate and (2) citrate dehydrase conversion of citrate to *trans*-aconitate, in the TCA cycle, adapted from Igamberdiev and Eprintsev [7].

In nature, aconitic acid exists in two forms: *trans* and *cis*, with the *trans*-aconitic acid (TAA) isomer being the predominant, stable form. Aconitic acid is produced by several higher plants as part of a strategy to balance the redox level, store fixed pools of carbon, ameliorate the impact of aluminum ions, and regulate reactions of the tricarboxylic acid (TCA) cycle [5,6,7]. The *cis* isomer of aconitic acid (CAA) is a low-level intermediate in the TCA cycle during the conversion of citrate to isocitrate by aconitase and can be converted to *trans*-aconitic acid, as shown in Figure 2. While the *trans*-form is energetically favored, the isomer conversion facilitated by the aconitate isomerase is reversible. On an industrial scale, aconitic acid can be abiotically synthesized from citric acid through a dehydration reaction with sulfuric acid or methane sulfonic acid [8].

While abiotically-synthesized aconitic acid is available, there is still considerable interest in producing or extracting aconitic acid inexpensively from renewable sources for use as chemical building blocks and precursors. Aconitic acid is the main organic acid that accumulates in sugar cane (*Saccharum officinarum* L.) and sweet sorghum (*Sorghum bicolor* L. Moench) and can be as high as 0.9–5.5% of dissolved solids [9]. It is present in the juice from the crushed sugar cane and sweet sorghum stalks as well as in the resulting syrups and molasses from the refining process [10]. In particular, TAA-rich sugar cane molasses is an inexpensive, renewable source of TAA. As a high-value chemical, TAA may provide another revenue stream for the sugar industry due to its industrial and biological applications [2].

Alternatively, one group has taken the approach of engineering *Escherichia coli* to produce aconitic acid through clustered regularly interspaced short palindromic repeats (CRISPR) interference-based modulation of metabolism pathways to produce high levels of aconitic acid using glucose as a carbon source for fermentation [11]. Moreover, another group heterologously expressed aconitate isomerase and aconitate hydratase in *E. coli* to produce TAA from citrate [12]. However, using sugar cane molasses or sweet sorghum syrup as a source of TAA may be more cost-effective and advantageous since these alternative renewable resources are readily available without the need for extensive genetic engineering or fermentation using expensive feedstock [2].

This review will focus on several different potential applications of aconitic acid in industry, its role in biological applications and systems, and the recovery of the acid from sugar crops. To the best of our knowledge, this is the first comprehensive review to cover aconitic acid research and its chemical and biological applications. The review spans from the earliest mention of aconitic acid in 1877 to the most recent relevant research in 2022. Seventy of the references are since 2010, 16 from 2000–2009, and 35 prior to the year 2000.

## 2. Industrial Applications

The potential use of aconitic acid in industry is impressive, often in the form of a reactant or precursor in an organic reaction. A summary of potential industrial applications of aconitic acid are listed in Table 1, which will be expanded on in the next sections.

### 2.1. Aconitic Acid Esters for Tissue Engineering

Aconitic acid can be widely used in the production of various polyesters due to the reactivity of its three carboxylic acid groups. Multiple examples are reported in the literature. For instance, aconitic acid has been used to produce biocompatible polyesters for bone tissue engineering. TAA, along with glycerol and cinnamic acid, were used to produce biocompatible polyesters through a polycondensation reaction to form a polymer scaffold that mimics extracellular matrix upon which bone tissue can be assembled in the presence of growth factors [15]. Additionally, thin layer polyester film scaffolds were synthesized to be applicable for skin tissue and wound healing with appropriate growth factors [15,16].

Cao et al. [13] reported the use of aconitic acid in the production of hyperbranched ester polymers. This was achieved by combining aconitic acid (B3 monomer) and di(ethylene glycol) (A2 monomer) in a one-pot polymerization reaction. The ester polymers were formed between the hydroxyl groups of the diethylene glycol and the three carboxylic acid groups of the aconitic acid, of which the third COOH group (farthest from the C-C double bond) designated c was the most reactive. This type of polymer may be useful for tissue engineering since it is biodegradable and compatible with tissues such as ophthalmic, cardiac, and vascular [13]. Another study utilized chemically produced citric acid-derived aconitic acid that could be decarboxylated to produce methylacrylic acid (MAA) using a BaAl_12_O_19_ catalyst to give a 51% yield [22]. MAA is useful in the production of biobased polymers [22].

### 2.2. Aconitic Acid Esters as Plasticizers

Plasticizers interact with polymers to increase flexibility and softness [26]. Phthalate acid esters (PAEs) such as di(2-ethylhexyl)phthalate (DEHP) are commonly added to polymers such as polyvinyl chloride (PVC) and to plastic films to improve flexibility and function [27,28]. However, many of these commonly used plasticizers are of environmental concern. For instance, plastic greenhouse films produced with PAEs are of particular concern because of their widespread use in commercial food production across the globe [29]. Generally, PAEs are not covalently bound to polymers and gradually disperse to pollute the air, soil, and fruits or vegetables produced in the greenhouse [30,31]. Leaching of PAEs from plastic films is a significant health and environmental safety concern [31,32]. In addition, PAEs are known endocrine disruptors linked to problems with reproduction, fetal development, and carcinoma, which further underscores the need for safe, bio-based plasticizers [33,34].

Because of the need for safe, bio-based plasticizers that do not contaminate or threaten the environment or human health, there has been a gradual shift toward harmless, plant-based options. Progress in bio-based plasticizers has been recently reviewed [17]. Similar to tri-butyl citrate, aconitic acid esters such as tri-butyl aconitate and tri-ethyl aconitate have also been reported as bio-based plasticizers [35,36,37]. In addition, Gilfillan and Doherty [18] reported that aconitic acid acts as a strong plasticizer for starch films. There are a few old U.S. patents (1942 and 1947) with limited information on the synthesis of aconitic acid esters [38,39].

### 2.3. Trans-Aconitic Acid as a Cross-Linking Agent

Multiple studies have reported that carboxylic organic acids such as malic acid and citric acid can be used as cross-linking agents for starch [40]. Another report by Gilfillan and Doherty [18] showed that aconitic acid, as an unsaturated tricarboxylic acid, is an effective cross-linking agent of starch polymer-based cast films at a low aconitic acid concentration of 2–5%. These films exhibited lower water solubility and a decreased swelling coefficient. At higher levels, aconitic acid acted as a strong plasticizer that reduced thermal stability and tensile strength, which produced a softer, more flexible film [18].

In addition, the carboxylic acid groups on TAA strongly react with amine groups. This reaction can be used to cross-link polybenzimidazole (PBI) chains with size-sieving ability to form a cross-linked polymer with improved separation capability. This has possible use in industrial H_2_/CO_2_ separation [21]. Similarly, another group also implemented TAA as a cross-linker in a poly-esterification reaction with ethylene glycol (ETG), but in microwave synthesis to form polyesters with varying degrees of cross-linking in under thirty minutes and without a solvent or catalyst [13,41].

### 2.4. Role in Microparticles and Grafting Agents

Aconitic acid has applications in the formation of polymers to produce microparticles and latexes. In one report, a polymer was made from aconitic acid and epoxidized linseed oil [19]. TAA has also been used to modify chitosan-based microparticles to improve solubility and bioavailability of salmeterol and fluticasone drugs for lung delivery [20]. The carboxylic acid groups of TAA can help improve the hydrophilicity of chitosan particles as well as swelling, which can affect drug delivery [20].

*Trans*-aconitic acid has been reported as a grafting agent for the modification of chitosan. Chitosan is a polymer derived from the deacetylation of chitin and can be modified with various functional groups that can be readily tailored to adsorption and removal of different pollutants such as pharmaceuticals from wastewater treatment facilities [24]. For instance, modification of chitosan with the carboxyl groups of TAA was used for the adsorption of diclofenac, a widely used anti-inflammatory pharmaceutical that pollutes water supplies [24].

### 2.5. Additional Aconitic Acid Uses in Green Chemistry

There are multiple studies incorporating forms of aconitic acid, especially trimethyl-*trans*-aconitate, in “green chemistry” reactions. For instance, trimethyl *trans*-aconitate can be used in an aza-Michael reaction with primary amines as a “green” click reaction to produce “tetra-functional N-alkyl- bis-(pyrrolidone dimethylcarboxylate)” as a monomer for polymer reactions [23]. Furthermore, aconitic acid, along with other polycarboxylates such as itaconic, fumaric, and malic acids, can be used to produce green surfactants by addition reactions [25]. Okada et al. [25] employed trimethyl *trans*-aconitate and alkyl mercaptan in an addition reaction to synthesize a polycarboxylate-based surfactant with S-type linkages that was biodegradable and capable of calcium sequestration [25].

The conversion of aconitic acid to itaconic acid, a C-5 dicarboxylic acid, and another Top 30 value-added chemical listed by the U.S. Department of Energy, can be chemically synthesized [1]. However, while the conversion of citrate to aconitic acid by dehydration followed by decarboxylation to itaconic acid is chemically feasible, it is economically unfavorable [8,42]. Therefore, from a cost perspective, it is more advantageous to produce bio-based itaconic acid from hydrolyzed lignocellulosic biomass via microbial fermentations discussed below.

## 3. Biological Roles of Aconitic Acid with Applications in Biological Engineering and Sustainable Agriculture

There are several reports of biological functions or roles of aconitic acid in organisms, including plants and microbes. These have implications for the production of value-added chemicals, agriculture, and biological engineering, which are discussed below and summarized in Table 2.

### 3.1. Microbial Conversion of Aconitic Acid to Itaconic Acid

The production cost of itaconic acid remains high. However, economic feasibility may be improved through optimization of either fermentation or chemical synthesis methods. Some options may include using sugarcane molasses, a sugar- and aconitic acid-rich feedstock, for chemical or enzyme-based decarboxylation or for improved fermentation conditions. Optimized fermentation conditions may include the use of inexpensive feedstocks such as agricultural waste products or through metabolic engineering of microbial strains to eliminate unwanted pathways and directly increase carbon flux toward itaconic acid production.

Some fungi naturally convert aconitic acid to itaconic acid through decarboxylation. For example, *Aspergillus terreus* is used in fermentations to produce itaconic acid [43,44,45,71]. In *A. terreus,* CAA is first transported by a mitochondrial transporter, At_MttA, from the TCA cycle in the mitochondria to the cytosol, where CAA is decarboxylated to itaconic acid by the *cis*-aconitic acid decarboxylase Cad-A [43,46,72]. Subsequently, itaconic acid is transported out of the mycelia by a specific transporter, belonging to the major facilitator superfamily (MFS) type transporter (MfsA) [73]. The genes involved in itaconic acid production in *A. terreus* also include a transcription factor, and the four genes are termed the “itaconate gene cluster” [73]. However, in the corn smut basidiomycete, *Ustilago maydis,* the mitochondrial transporter Um_Mtt1, also transports CAA from the mitochondria to the cytoplasm, but CAA is first isomerized to TAA by aconitate-Δ-isomerase, Adi1, then decarboxylated by *trans*-aconitic acid decarboxylase, Tad1, to itaconic acid [43,46,47,74]. The “itaconate gene cluster” in *U. maydis* is induced upon nitrogen limitation [75]. Overall, the metabolic differences in itaconic acid production by these two fungi is notable because it demonstrates the possibility of producing itaconic acid from either the *trans* or *cis* isomer of aconitic acid in different organisms [47]. Differential microbial conversions of CAA and TAA to itaconic acid may also provide insight into ex vivo enzymatic conversion options with recombinantly expressed decarboxylases.

### 3.2. Microbial Use as a Carbon Source

Microorganisms such as the soil bacterium *Pseudomonas* sp. WU-0701 encodes an aconitate isomerase that catalyzes the reversible isomerization between TAA and CAA, an intermediate in the conversion of citrate to isocitrate in the TCA cycle [49]. This enables the organism to grow on TAA as a sole carbon source by isomerizing TAA to CAA, which feeds back into the TCA cycle. Interestingly, others have reported the presence of various *Pseudomonas* species in the rhizosphere of sugar cane, while others have reported that some rhizosphere-associated bacteria may improve plant growth and photosynthesis under certain conditions [76]. The utilization of TAA as a carbon source by some of these bacteria could suggest a possible symbiosis between sugar cane and microorganisms in the rhizosphere involving TAA.

### 3.3. Aconitic Acid as a Fermentation Inhibitor

There are some reports that aconitic acid may act as an inhibitor in the fermentation of sugar cane juice, syrup, molasses, and sweet sorghum syrup. When studying the fermentation of sweet sorghum juice as a function of harvesting time, Day and Sarkar [77] noted that the yield of ethanol produced by sake and wine yeasts dropped later in the harvest season, even though the sugar content increased. They speculated that the aconitic acid was responsible for the reduction in ethanol yield. When increasing the fermentable sugars by concentrating sweet sorghum juice, Wu et al. [78] reported that the fermentation efficiency decreased with increasing water removal and speculated that the increase in aconitic acid levels due to the water removal may have been the reason [78]. In fermentation of sweet sorghum juice, Gibbons and Westby [79] reported low ethanol yields by *Saccharomyces cerevisiae* depending on sweet sorghum variety. They speculated that aconitic acid was partially responsible for the inhibition, as it varied between varieties. The inhibition also remained noticeable in the fermentation of mixtures of sweet sorghum juice with hydrolyzed cereal mash. More inhibition was noted with corn mash than with wheat mash. Another report showed that the fermentation rate decreased by 29% when comparing the production of ethanol from sweet sorghum juices containing 0.114 and 0.312% aconitic acid [50]. These authors attributed the rate reduction to aconitic acid and showed that the intracellular acid concentration of the yeast increased by a factor of 2 and 4 when the pH is changed from pH 5.0 to pH 3.5 to pH 2.0. In a detailed study, Klasson [52] showed that it was the undissociated form of aconitic acid that was responsible for inhibition of ethanol production by Distiller’s yeast, *S. cerevisiae*, in the fermentation of sweet sorghum sugars. By controlling the pH during the fermentation, the inhibition could be overcome, and when the pH was controlled above 4.5, the presence of aconitic acid (5 g/L) became slightly advantageous, and ethanol titer (+4%) and yield (+3%) increased slightly, confirming results of a previous study in synthetic media [51]. At a fermentation pH > 4.5, the fermentation of diluted sweet sorghum syrup to butanol by *Clostridium beijerinckii* did not appear to be inhibited by aconitic acid [52].

### 3.4. Nematocidal Activity of Trans-Aconitic Acid

Pathogenic nematodes can be a significant problem for some crops such as sugar beets and cotton. For instance, the beet cyst nematode, *Heterodera schachtii*, is especially problematic for sugar beets, which supply about one-third of the world’s supply of sugar [80]. Finding a sustainable, plant-based source of nematocide that is nontoxic to humans is of considerable interest. Interestingly, the soil bacterium, *Bacillus thuringiensis,* produces TAA as a virulence factor against soil nematodes [53]. Studies with the thuringiensin-producing strain, *B. thuringiensis* CT-43 in particular, revealed a chemical product called CT-A, with nematocidal activity against the major pest root-knot nematode, *Meloidogyne incognita* [53]. Further study revealed that CT-A contains TAA and that TAA exhibits a significantly higher nematocide activity than the *cis* isomer, CAA, in a survival bioassay with *M. incognita* J2s after 72 h. A plasmid-encoded operon for TAA biosynthesis was described in *B. thuringiensis* CT-43 that encodes an aconitate isomerase, named TAA biosynthesis-related gene A (tbrA), and a membrane-bound transporter *tbrB* that transports TAA out of the cell [53,81].

### 3.5. Anti-Leishmanial Activity of Trans-Aconitic Acid

Anti-leishmanial activity has also been attributed to TAA against the protozoan pathogen, *Leishmania donovani,* the causative agent of visceral leishmaniasis, also known as kala-azar, thirty years ago, which is potentially fatal and difficult to treat [54,55]. During the lifecycle of this protozoan, the promastigote form is present in the vector during disease transmission, while the amastigote form is found intracellularly within infected macrophages of the host. Anti-leishmanial drugs can be problematic due to toxicity. TAA was investigated as an alternative and in combination with conventional chemotherapy since TAA is an inhibitor of the aconitase enzyme in the TCA cycle [56]. The *L. donovani* amastigote relies on mitochondrial β−oxidation of fatty acids as an important energy source. During β-oxidation, fatty acids are converted to acetyl-CoA, which feeds into the TCA cycle to generate ATP for energy, so TAA was of particular interest as an inhibitor of aconitase in the TCA cycle [57]. Interestingly, 20 mM TAA significantly attenuated promastigote replication that could be reversed by the addition of 20 mM CAA at 72 h, indicating divergent biological activity of the two aconitic acid isomers. Furthermore, 2 mM TAA reduced parasitic liver burden in infected hamsters in a dose-dependent manner [54,55]. A dose of 2 mM TAA reduced the number of amastigotes within a macrophage model by 60% [54]. Five (5) mM TAA, together with anti-leishmanial drugs, sodium stibogluconate, pentamidine, or allopurinol, completely inhibited amastigote transformation to promastigote [55]. These reports from *L. donovani* may provide insight into the mechanism of action against other organisms such as nematodes.

### 3.6. Aconitic Acid Production Confers Survival Advantages

The production of TAA by sugarcane, sweet sorghum, and other plants may confer a survival advantage against pests and help to regulate metabolic processes during rapid plant growth. Stout et al. [82] assayed 94 species of grasses and non-grasses from rangeland and found that 47% of grasses and 17% of non-grasses accumulated TAA to high levels. Furthermore, aconitic acid has also been detected in grasses such as oats, rye, wheat, barley, and maize [83].

In higher plants, *trans*-aconitate is produced and stored as a “tricarboxylic acid pool” [7]. TAA is produced via two mechanisms connected to the TCA cycle. The first occurs via the citrate valve and citrate hydratase to form TAA [7]. The second mechanism occurs with aconitase conversion of citrate to isocitrate via a *cis*-aconitate intermediate, which can then be isomerized to TAA via aconitate isomerase [7]. Accumulation of TAA may play a role in regulating the TCA cycle by inhibition of aconitase [56]. Moreover, this inhibition can be alleviated by monomethyl esterification by *trans*-aconitate methyltransferase TMT1, which has been described in *Escherichia coli*, *Saccharomyces cerevisiae*, and *Ashbya gossypii* [84,85,86].

#### 3.6.1. Antifungal Defense

Aconitic acid may also play a role in antifungal defense in some plants. For example, TAA may accumulate in wheat as part of a protective mechanism against powdery mildew *Blumeria graminis f.* sp. *Tritici* [58]. In particular, TAA and, to a much lesser extent, CAA can be induced to high levels of accumulation in wheat leaves by potassium sulfate [87]. Moreover, later studies showed that in wheat plants experimentally infected and fed silicon, TAA was methylated to form methyl TAA, which acts as a phytoalexin to limit disease [58].

Organic acid root exudates such as malic and tartaric acid also appear to inhibit fusarium wilt, caused by *Fusarium oxysporum f.* sp. *fabae* (FOF), in faba beans (*Vicia faba*). TAA was only detected in root exudates under nitrogen limitation, while tartaric and malic acids were detected after nitrogen application. So far, it is unknown what role TAA may play in faba bean antifungal defense during limited nitrogen conditions [88].

#### 3.6.2. Antifeedant

TAA also appears to function as an antifeedant in some plants, such as barnyard grass against the brown planthopper (*Nilaparvata lugens*) [59]. Additional studies further demonstrated the resistance of barnyard grass and one resistant strain of rice “Babawee” to feeding by brown planthopper due to the presence of TAA, but not CAA. Furthermore, TAA was not detected in the susceptible rice strain “Koyonishiki” [59,60,61,62]. High levels of aconitic acid production may also play a role in the resistance of some cereal plants such as corn, sorghum, and barnyard grass to aphids [89]. For instance, higher levels of TAA in sorghum leaves corresponded to decreased aphid burden and leaf damage, further implying that TAA functions as a defensive phytochemical [89,90,91].

#### 3.6.3. Defense against Aluminum Toxicity

Aconitic and oxalic acids are the predominant organic acids produced in maize. Aconitic acid appears to protect the maize against aluminum toxicity. The level of organic acids in maize is high during early harvest and decreases with each successive harvest [5]. About 60% of the aconitic acid is the *trans* isomer [5]. TAA is found in both the shoots and roots of maize and may help protect the plant from Al toxicity through organic acid chelation [6]. TAA was found to accumulate in the roots to higher levels in response to Al^3+^ activity than in the shoots [6].

Interestingly, since TAA-producing grasses such as sugar cane are often part of a crop rotation strategy with soybeans, and since sugar cane vinasse is sometimes applied to fields, the effect of TAA on soybean growth was investigated. TAA was found to inhibit soybean growth by inhibiting photosynthesis and increasing H_2_O_2_ in roots, which resulted in decreased water uptake [69]. It is unknown whether any residual TAA remains in the soil after harvesting sugar cane or whether TAA rapidly dissipates to negligible levels, but it is worth considering the possible impact of TAA on crop rotations with soybean.

### 3.7. Biofilm Inhibition

Aconitic acid may be an inhibitor of biofilm formation. Pestana-Nobles et al. [70] reported a computational study based on molecular docking and molecular dynamic simulation that screened 224,205 molecules from the natural products ZINC15 database. The results predicted TAA as a possible ligand and inhibitor of the PleD protein involved in bacterial biofilm formation. PleD and its homologs are diguanylate cyclases that contain a GGDEF domain involved in cyclic di-GMP second messenger formation, a critical signaling molecule involved in quorum sensing needed for biofilm formation. As such, PleD homologs are often the target of high-throughput screens for biofilm inhibitors [92,93]. While TAA was identified computationally as an inhibitory ligand for PleD, to our knowledge, it has yet to be experimentally validated.

### 3.8. Anti-Inflammatory Treatment

TAA has also been reported as an anti-inflammatory treatment for conditions such as arthritis with mucoadhesive microspheres containing TAA [63,64]. For instance, the medicinal plant *Echinodorus grandifloras* contains high levels of TAA and is used to treat rheumatoid arthritis in Brazil. TAA, along with other fractions extracted from *Echinodorus grandiflorus* leaves, acts as an anti-inflammatory by inhibiting tumor necrosis factor-alpha (TNF-α) release during in vitro assay of lipopolysaccharide (LPS)-stimulated, THP-1 human monocyte cells [65]. Furthermore, the lipophilicity of TAA can be improved by Fisher esterification with an alcohol to form mono- di- or tri-esters of TAA [64]. Improving lipophilicity of TAA by esterification was used as a strategy to improve the pharmacokinetics and transport of TAA across biological membranes [94]. The TAA esters were administered orally and tested in a mouse model of lipopolysaccharide (LPS)-induced arthritis. TAA diesters were found to be the most biologically active, and the anti-inflammatory activity increased the longer the aliphatic chain of the alcohol was used for esterification [64].

### 3.9. Antioxidant Activity

TAA has also been reported to function as an antioxidant. For instance, TAA is present in *Syzygium polyanthum*, a plant used in Indonesia as a spice. Plant extracts from *S. polyanthum* containing TAA exhibited DPPH (2,2-diphenyl-1-picrylhydrazyl) antioxidant activity [66,67]. TAA has also been used as a model antioxidant in the development of lecithin-based, antioxidant-loaded nanoliposomes [68].

## 4. Aconitic Acid in Sugar Cane and Sweet Sorghum and Its Recovery

As previously mentioned, TAA is the main organic acid that is naturally present in sugar cane [95]. Its presence in sugarcane juice was described as early as 1877 [96] and in sweet sorghum in 1882 [97], and its decrease during sugarcane development has been proposed as a maturity indicator, decreasing with sugarcane maturity [98].

### 4.1. Aconitic Acid Changes during Plant Development

**Figure 3:**
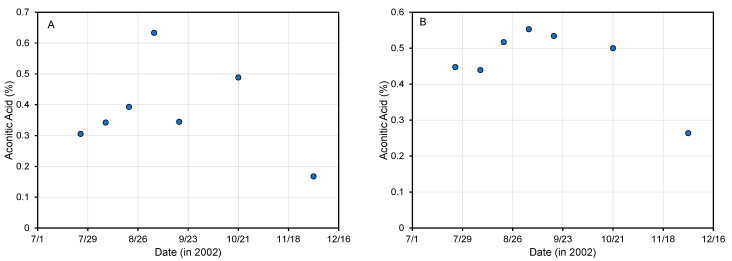
*Trans*-aconitic acid in sugarcane stalk juice (**A**) and in water extract from tops/leaves (**B**). Adapted from Gil Zapata [27].

The changes during the season in sugarcane stalk juice and extract from tops/leaves are shown in Figure 3. The limited data show a possible increase in aconitic acid in sugarcane juice during the growing season (July to mid-September) and a decrease during the harvesting season (mid-September to January). The Pearson Product Moment Correlation showed a statistically significant (*p* = 0.03) correlation (0.80) between the aconitic acid in the juice and cane tops/leaves. According to Gil Zapata [27], the aconitic acid in the sugarcane leaves is approximately 3–6 times higher than in stalk juice, which is consistent with information reported by others [99]. Ripener had no impact on aconitic acid in stalk juice but did have an impact in tops/leaves, where those plants treated with ripener had less aconitic acid [27]. This is consistent with the general understanding that aconitic acid decreases after maturity [98].

**Figure 4:**
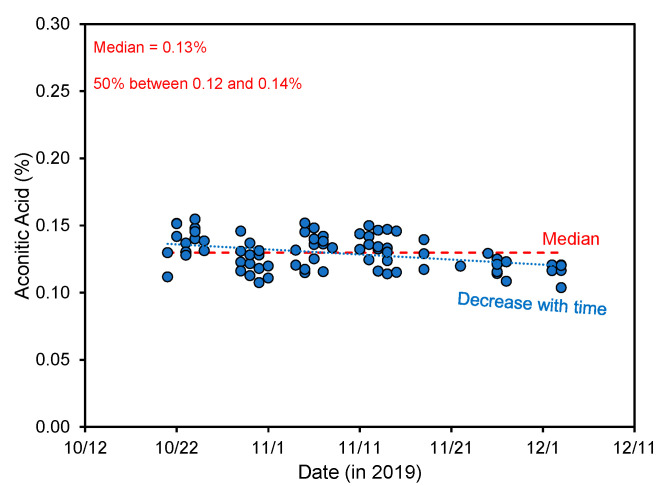
Concentration of aconitic acid in crusher juice during the 2019 Louisiana harvesting season from one raw sugar factory (data by the authors). The aconitic acid was measured by high-pressure chromatography as previously described [10], after centrifugation, filtration (0.45 µm pore size), and 6× dilution.

During an extended sampling campaign during the 2019 harvesting season in Louisiana, USA, the aconitic acid concentration was very consistent in raw sugar factory crusher juice with a median of 0.13%, but statistically (*p* < 0.001) decreased slightly during the harvesting season, as shown in Figure 4.

### 4.2. Impact of Plant Cultivar and Growth Location on Aconitic Acid Content

There is evidence that aconitic acid in sugar cane is impacted by cultivar and growing region [100]. For example, Gil Zapata [27] reported that variety L 97-128 had less than half the aconitic acid in tops/leaves when compared with cultivars LCP 85-384 and HoCP 91-555. Among the cultivars, HoCP 96-540, HoCP 04-838, HoCP 09-804, and L 01-299; HoCP 04-838 had significantly more *trans*-aconitic acid in the sugarcane juice (0.17%) when compared to the other cultivars [101,102], while HoCP 09-804 had more *cis*-aconitic acid than the others [102]. When a commercial process was developed for the recovery of aconitic acid from sugarcane streams, others noted that the sugarcane varieties were not grown for their high aconitic acid content but could be cultivated if so desired, at the expense of sucrose [99]. Whether or not varietal differences were the source of variation, it was reported that molasses from sugarcane grown in Louisiana contains more aconitic acid than molasses from the tropical climate of Guadalupe [103]. Martin [104] also noted that tropical molasses contained lower amounts of aconitic acid when compared to Louisiana molasses. Furthermore, Haines Jr. and Joyner [99] attributed the higher aconitic acid content in Louisiana sugar cane molasses to the shorter growing season when compared to Florida-grown cane. Summer fallow had no significant impact on the aconitic acid concentration in sugarcane juice [102].

Almodares et al. [105] noted that both cultivar and maturity impacted aconitic acid concentration in sweet sorghum, where a greater maturity resulted in lower aconitic acid concentration in the plants. They also noted that if nitrogen fertilization was distributed throughout the maturity, the aconitic acid concentration was less than if nitrogen was only applied at planting. They inferred that aconitic acid was better consumed in the TCA cycle when nitrogen was continuously supplied [105]. When studying aluminum toxicity, others found that cultivars tolerant to aluminum accumulated more aconitic acid in roots and leaves and that the cation most likely regulated some enzymes causing both malic and aconitic acid to accumulate when exposed to increased levels of aluminum [106]. It was suggested that higher levels of these organic acids could internally be involved in a detoxification mechanism, just as in the case of maize [6]. Others noted a strong relationship between planting date and aconitic acid content in the sweet sorghum juice at the hard-dough maturity stage in 23 cultivars and hybrids [107]. Later planting dates (April, May, or June) resulted in higher aconitic acid concentrations at the same maturity. There was also a strong correlation with the plant variety. Among the cultivars, N98 had the highest average juice aconitic acid content (April–June, plantings) of 0.32%. The average among all the 199 juice samples (variety, planning date, replicates) collected was 0.20%, with a range of 0.0–0.58%. In plantings over two years, there was no statistical difference in the aconitic acids in the same 23 cultivars and hybrids [108]. In another study that spanned two years and 41 samples, the average concentration of aconitic acid in sweet sorghum juice was 0.30%, with a range of 0.026–0.56% [50].

### 4.3. Fate of Aconitic Acid during Sugar Processing

In the production of sugar from sugar cane, aconitic acid survives the clarification, evaporation, and crystallization operation during normal sugarcane juice processing and is present in the molasses [109]. The only significant loss of aconitic acid occurs in the scales and in the deposits of evaporators, which can contain 8% aconitic acid [100]. Of the normal sugar processing by-products, molasses contains the most aconitic acid, where the concentration can be as high as 4.4% (based on Brix) in C (Backstrap) molasses [109]. If the molasses contains significant insoluble solids, these solids can contain 16–35% aconitic acid [109] or even over 50% [100]. A recent report indicates that some of the TAA remains in the unrefined cane sugar (i.e., raw sugar) as an “unambiguous metabolite” detectable by ^1^H-NMR spectroscopy, which can be used for differentiation from other sugar products in foods and useful to detect sugar adulterations [110]. In the processing of sweet sorghum juice to syrup, the aconitic acid also survives the clarification and evaporation process, accumulating in the syrup, where the concentration can be as high as 2.8% in unfiltered samples or 1–1.6% in filtered syrup samples [10,111]. Therefore, just as in the case of sugarcane juice processing, the evaporator scales in sweet sorghum juice processing contain aconitic acid [97].

### 4.4. The Recovery of Aconitic Acid from Sugar Crops

The recovery of aconitic acid from sugarcane molasses has been performed both on a laboratory and commercial scale. Kanitkar et al. [2] reported up to a 69% recovery of TAA from Louisiana sugarcane molasses with a 99.9% purity using ethyl acetate extraction. Moreover, various liquid-liquid extraction methods have been reported, including those with butanol [27]. The extraction efficiencies were examined and found to yield varying aconitic acid quantity and purity; for instance, butanol extraction yields more aconitic acid but lower purity [15]. Therefore, extraction methods should be carefully weighed with consideration of downstream applications that may require either high purity or high yield. The recovery of aconitic acid from sugarcane and sweet sorghum juice, syrup, and molasses via precipitation has been patented [112,113,114], as well as a process using ion exchange [115,116]. The precipitation method from sugarcane molasses was once the only method by which dicalcium magnesium aconitate was produced for plasticizer use [99]. The process included dilution, addition of lime and calcium chloride, followed by centrifugation to recover the precipitate, which was washed, centrifuged, and dried. At the time, Louisiana backstrap molasses could have served as a source of 10,000,000 pounds of aconitate, if the process had been fully implemented.

It was also reported that aconitic acid, as well as other organic acids, is present in stillage (vinasse) from fermented and distilled sugar cane juice and molasses during ethanol production [103]. Among the organic acids, part of the aconitic acid also remained in the effluent from the anaerobic digestion of rum vinasse as part of the waste treatment.

### 4.5. Aconitic Acid Recovery as Part of Fermentation of Sugars

In laboratory studies, Gil Zapata [27] fermented juice from sugar cane tops to ethanol, extracted the ethanol via evaporation, removed the solids, and attempted to recover the TAA through ion exchange. Two of the adsorbents were able to remove 86–88% of the aconitate from the pH 5.1 stillage. A third adsorbent was identified as suitable for aconitic acid recovery at low pH. Ultimately, the use of the non-ionic Dowex Optipore SD-2 adsorbent was recommended at pH 2.8 and TAA recovery from the adsorbent with butanol at elevated temperatures. A weak anionic resin (sulfate form) was used to remove TAA from a sugar cane-based ethanol distillery by others, where it was noted that while the aconitate can be removed at stillage pH 4.5, a significant amount of inorganic anions and other materials were also removed leading to only 39% purity in the purified extract [117]. The elution from the anionic resin column was done with weak sulfuric acid.

Recovery of aconitic acid after fermentation of sugar cane molasses but before distillation was studied by Madsen Kanitkar et al. [2], who used ethyl acetate to extract aconitic acid from spent, centrifuged, and filtered molasses fermentation products. Impressively, the aconitic acid yield and purity of the material extracted from unfermented and fermented diluted molasses were very similar. The authors suggested that TAA extraction of fermented molasses would be preferred as ethanol is also generated [2]. Ethyl acetate was also the selected solvent for TAA extraction from ethanol production vinasse by others, who extracted the vinasse TAA at pH 2.0 with solvent, evaporated the solvent to form a solid phase, which was dissolved in water, and then contacted with an anion exchange column to separate the TAA with 90% purity [118]. After additional purification steps, including color removal, methylation, chromatography, and hydrolyzation, 99% purity of TAA was obtained.

While the above work focused on the recovery of TAA during different steps of fermentation of sugar cane to ethanol, similar recovery systems may also apply for other sugar feedstocks (e.g., sweet sorghum) and other fermentation systems. Examples of fermentation products that were produced from sweet sorghum syrup include succinic acid, acetone/butanol/ethanol, and acetoin, where aconitic acid was present before and after fermentation (see Table 3). From these results, it appears that succinic acid, acetoin, and ethanol fermentations retain aconitic acid in the spent fermentation broth, while the acetone/butanol/ethanol fermentation depleted aconitic acid in the broth.

## 5. Conclusions

In summary, recovered aconitic acid from sugar cane and sweet sorghum waste products could also be further utilized for ex vivo enzymatic reactions using recombinantly expressed aconitate decarboxylase enzymes, either from *A. terreus* or *U. maydis*, for conversion of aconitic acid to itaconic acid or other high-value chemicals. The numerous biological roles of aconitic acid are promising for the further development of bio-based products or strategies ranging from pharmaceutical treatments to sustainable agriculture practices. For instance, the survival advantage conferred by aconitic acid to some plants may also inform future genetic engineering approaches aimed at improving resistance to fungal disease and various crop pests. It is also conceivable that sugar cane or sweet sorghum could be genetically modified for higher aconitic acid production to produce strains tailored for downstream industrial or biological applications. Furthermore, the metabolic engineering of microbes such as bacteria or fungi to produce high levels of aconitic acid from inexpensive feedstock could also be advantageous.

In addition, since sugar cane milling only occurs three months out of the year in subtropical Louisiana, in the remaining months, molasses or vinasse could be used for recovery of aconitic acid for alternative streams of income. By pairing low-cost, renewable sources of aconitic acid with high-value applications related to industrial and biological purposes, there is vast potential for cost-effective production of high-value aconitic acid-derived chemicals and bio-based products, thereby providing additional income streams to the sugar industry.

## Figures and Tables

**Table 1:** Summary of potential industrial applications of aconitic acid.

Industrial Uses and Applications	References
formation of polyesters for tissue engineering	[13,14,15,16]
bio-derived plasticizer	[17,18]
hyperbranched ester polymers	[13]
chemical conversion to C5 itaconic acid	[1]
polymers to form microparticles for drug delivery	[19,20]
cross-linking of polybenzimidazole chains for H_2_/CO_2_ separation	[21]
cross-linking of starch polymers	[18]
production of methylacrylic acid	[22]
*trans*-tri-methyl aconitate in green click reactions	[23]
grafting agent to modify chitosan as an adsorbent	[24]
production of green surfactant	[25]

**Table 2:** Reported biological roles of aconitic acid.

Biological Uses and Applications	Method or Approach	References
microbial production of itaconic acid	*Aspergillus terreus* decarboxylation of CAA	[43,44,45]
microbial production of itaconic acid	*Ustilago maydis* decarboxylation of TAA	[43,46,47,48]
*Pseudomonas* sp. use as sole carbon source	isomerization of TAA to CAA for TCA cycle	[49]
fermentation inhibitor	in *Saccharomyces cerevisiae*, pH-dependent	[50,51,52]
nematocidal activity	*Meloidogyne incognita*	[53]
anti-leishmanial activity	*Leishmania donovani*	[54,55]
regulation of TCA cycle	TAA-based inhibition of aconitase	[7,56,57]
antifungal defense in plants	methyl-TAA acts as a phytoalexin	[58]
antifeedant	involved in resistance of some plants to *Nilaparvata lugens*	[59,60,61,62]
defense against aluminum toxicity	organic acid chelation of Al	[5,6]
anti-inflammatory activity	inhibition of TNF-α release by monocytes	[63,64,65]
antioxidant activity	DPPH assay and nanoliposomes	[66,67,68]
inhibitor of Glycine max	Increased H_2_O_2_ in roots and reduced water uptake	[69]
inhibitor of quorum sensing	ligand inhibitor of PleD	[70]

**Table 3:** Aconitic acid before and after fermentation to produce different products from diluted sweet sorghum syrup. (The original references may not contain the aconitic acid concentrations but have been added here.).

Fermentation	Aconitic Acid before Fermentation	Aconitic Acid after Fermentation
Succinic acid using *Escherichia coli* AFP 184 [119]	0.11%	0.072%
Acetone/butanol/ethanol using *Clostridium beijerinckii* NCP 260 [10]	0.075% (Syrup a) 0.082% (Syrup b)	0.001% (Syrup a) 0.002% (Syrup b)
Acetoin using *Bacillus subtillus* NFRI 8291 and NFRI 8299 [120]	0.304%	0.325%
Ethanol using Baker’s yeast [121]	0.28% (Clarifier mud)	0.25%
